# The factor structure of the twelve item General Health Questionnaire (GHQ-12): the result of negative phrasing?

**DOI:** 10.1186/1745-0179-4-10

**Published:** 2008-04-24

**Authors:** Matthew Hankins

**Affiliations:** 1 King's College London, Department of Psychology (at Guy's), Institute of Psychiatry, London, UK; 2 Department of Primary Care & Public Health, Brighton & Sussex Medical School, Brighton, UK; 3 Brighton & Sussex University Hospitals NHS Trust, Royal Sussex County Hospital, Brighton, UK

## Abstract

**Background:**

The 12-item General Health Questionnaire (GHQ-12) is used routinely as a unidimensional measure of psychological morbidity. Many factor-analytic studies have reported that the GHQ-12 has two or three dimensions, threatening its validity. It is possible that these 'dimensions' are the result of the wording of the GHQ-12, namely its division into positively phrased (PP) and negatively phrased (NP) statements about mood states. Such 'method effects' introduce response bias which should be taken into account when deriving and interpreting factors.

**Methods:**

GHQ-12 data were obtained from the 2004 cohort of the Health Survey for England (N = 3705). Following exploratory factor analysis (EFA), the goodness of fit indices of one, two and three factor models were compared with those of a unidimensional model specifying response bias on the NP items, using structural equation modelling (SEM). The hypotheses were (1) the variance of the responses would be significantly higher for NP items than for PP items because of response bias, and (2) that the modelling of response bias would provide the best fit for the data.

**Results:**

Consistent with previous reports, EFA suggested a two-factor solution dividing the items into NP and PP items. The variance of responses to the NP items was substantially and significantly higher than for the PP items. The model incorporating response bias was the best fit for the data on all indices (RMSEA = 0.068, 90%CL = 0.064, 0.073). Analysis of the frequency of responses suggests that the response bias derives from the ambiguity of the response options for the absence of negative mood states.

**Conclusion:**

The data are consistent with the GHQ-12 being a unidimensional scale with a substantial degree of response bias for the negatively phrased items. Studies that report the GHQ-12 as multidimensional without taking this response bias into account risk interpreting the artefactual factor structure as denoting 'real' constructs, committing the methodological error of reification. Although the GHQ-12 seems unidimensional as intended, the presence of such a large response bias should be taken into account in the analysis of GHQ-12 data.

## Background

The 12-item General Health Questionnaire (GHQ-12) is a self-report measure of psychological morbidity, intended to detect "psychiatric disorders...in community settings and non-psychiatric settings" [1]. It is widely used in both clinical practice [2], epidemiological research [3] and psychological research [4].

The GHQ-12 has been extensively evaluated in terms of its validity and reliability as a unidimensional index of severity of psychological morbidity [5-9]. Many studies, however, have reported that the GHQ-12 is not unidimensional, but instead assesses psychological morbidity in two or three dimensions [10-19]. Several two- and three-dimensional models have been proposed (see Martin & Newell 2005 for review [20]), and to date no study examining the factor structure of the GHQ-12 has found it to be unidimensional. These various factors have been interpreted as substantive psychological constructs such as 'Anxiety', 'Psychological distress', 'Social Dysfunction', 'Positive Health', for example. When these competing models have been compared [10,20], confirmatory factor analysis suggests that the best fitting model is the three-dimensional model of Graetz [21], which proposes that the GHQ-12 measures three distinct constructs of 'Anxiety', 'Social dysfunction' and 'Loss of confidence'.

The GHQ-12 was validated on the assumption that it was a unidimensional and generic measure of psychiatric morbidity: if it truly is multidimensional, measuring psychological functioning in three specific domains, then the validation data may be questioned, and hence the use of the GHQ-12 in clinical practice and research contexts.

Despite the apparently consistent evidence that the GHQ-12 is multifactorial, the findings of these studies could be interpreted as demonstrations of a phenomenon long-known in the psychometric literature: that measure comprising a mixture of positive and negative statements can produce an entirely artefactual division into factors [22-27]. Such artefacts, introduced as they are by the method of measurement, are known as 'method effects'.

The GHQ-12 itself comprises six items that are positive descriptions of mood states (e.g "felt able to overcome difficulties") and six that are negative descriptions of mood states (e.g. "felt like a worthless person"). For brevity these will be referred to as 'negatively phrased items' (NP items) and 'positively phrased items' (PP items) respectively. Consistent with the hypothesis that these factors derive from method effects, a casual inspection of the two and three factor structures previously reported reveals that they comprise most (or all) of the NP items and most (or all) of the PP items. For example, of the four two factor solutions included in the review by Newell & Martin, two comprise all of the NP items vs. all of the PP items, and two comprise all of the NP items plus one PP item vs. the remaining PP items. Of the three three factor solutions reviewed, the additional factor comprises either two NP items or two PP items, i.e. the division between NP and PP items is maintained. In addition, when reported, the correlations between dimensions are very high; this is again consistent with the hypothesis that the GHQ-12 is unidimensional, and that the apparent two- or three-dimensional structure is artefactual. Hence the question arises of whether the factors identified in these studies truly reflect clinically distinct dimensions of psychological morbidity. If the dimensions identified by these studies are simply due to the measurement bias introduced by method effects, then the constructs identified do not exist (a methodological error known as 'reification' [28]).

The analyses so far reported employed either exploratory factor analysis (EFA) to derive the factor structure or confirmatory factor analysis (CFA) to confirm the factor structure of the GHQ-12. EFA cannot in principle distinguish between genuine multidimensional structure and spurious factors generated by method effects. CFA, used appropriately, can model such method effects and so test the hypothesis that the apparent factor structure is artefactual. Unfortunately, studies employing CFA have only tested the models originally generated by EFA; used in this way, CFA is also in principle incapable of distinguishing between a genuine factor structure and an artefactual one.

The modelling of the proposed method effect is, however, relatively straightforward. Various mechanisms have been proposed for the general phenomenon of NP and PP items separating into factors, focusing on respondents' difficulties in processing negatively-phrased items. It has been suggested that negatively phrased items are more difficult to process because of inattention, variation in education, or an aversion to negative emotional content [22,24,25]. Whatever the cause, since response bias introduces variation in responses not due to variation in the measured construct, nor to random measurement error (both of which should apply equally to all items), then it may be hypothesised that response bias creates additional variation in negatively-phrased item scores, and that this additional variance is common to the negatively phrased items but not the positively phrased items. Two hypotheses follow from this: (1) NP items should have significantly higher variance than PP items and (2) a unidimensional model incorporating response bias on the NP items should fit the data better than the two- or three-factor models proposed.

The aim of this study was therefore to explore the possibility that the previously-reported factor structures of the GHQ-12 were due to method effects, namely a response bias on the negatively phrased items.

## Methods

GHQ-12 data were obtained from the 2004 cohort of the Health Survey for England, a longitudinal general population conducted in the UK. Sampling and methodological details are in the public domain [29]. For the purposes of this study, a single adult was selected from each household to maintain independence of data. Of the 4000 such adults, 3705 provided complete data for the GHQ-12. The Likert scoring method was used, with each of the twelve items scored in the range 1 to 4, 4 indicating the most negative mood state. For clarity, the six NP items were labelled n1 to n6 and the six PP items p1 to p6.

The variances of all items were computed with 95% confidence limits, the hypothesis being that the variances on the negative questions would be uniformly greater than those on the positive questions. In addition, Pearson correlation coefficients were calculated between all items (producing a correlation matrix). Exploratory factor analysis was first conducted to explore whether the data would replicate either the two- or three-factor solutions previously reported; in the context of this study, this was a necessary step, since in the absence of a multifactorial solution there would be no method effect to explain. Consistent with most of the previous EFA analyses, the principal components method was used, with orthogonal (Varimax) rotation.

Following EFA, four models were tested for goodness of fit (CFA) using the structural equation modelling package AMOS 6.0 (maximum likelihood method):

1. Unidimensional: one factor, i.e. the intended GHQ-12 measurement model

2. Confirmatory analysis of the EFA solution

3. Three dimensional: three correlated factors, derived from the Graetz [21] model of three factors measured by 6 items ('anxiety', p1 to p6), 4 items ('social dysfunction', n1 to n4) and two items ('loss of confidence', n5 and n6)

4. Unidimensional with method effects: one factor (essentially Model 1) with correlated error terms on the NP items

**Figure 1:**
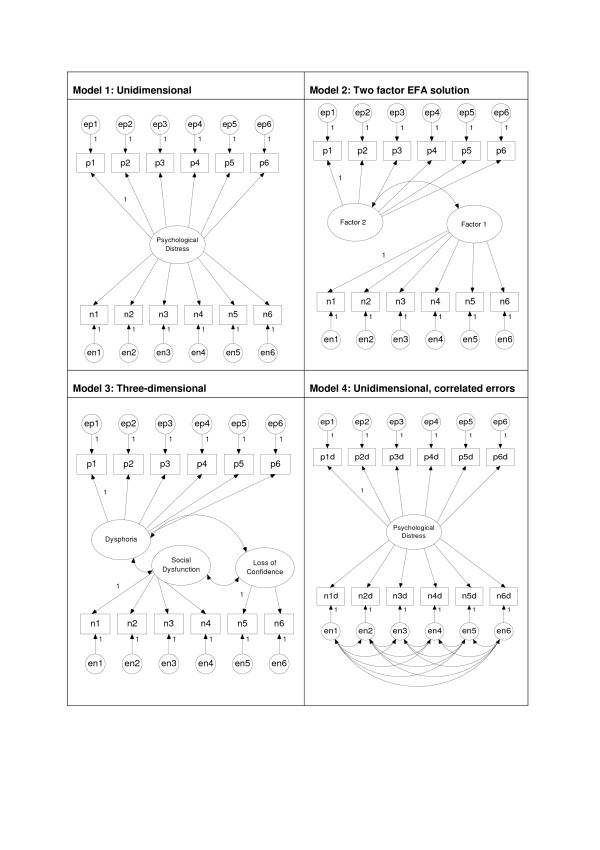
Model specification (models 1 to 4).

Models 1 to 3 are typical of the EFA/CFA approach taken to date. Model 4 is the model hypothesised to have the best fit for the data. Path diagrams for all models are presented in Figure 1. As recommended by Byrne [30] a range of goodness of fit indices were computed for model comparison. These indices differ primarily in their treatment of sample size, model parsimony and the range of values considered to indicate a well-fitting model. The goodness-of-fit index (GFI), adjusted goodness-of-fit index (AGFI), normed fit index (NFI) and comparative fit index (CFI) all increase towards a maximum value of 1.00 for a perfect fit, with values around 0.950 indicating a good fit for the data. In contrast, the values of the root mean square error of approximation (RMSEA) and the expected cross-validation index (ECVI) decrease with increasingly good fit, and are not limited to the range zero to one. The ECVI indicates how well the model will cross-validate to another sample, while the RMSEA provides a 'rule of thumb' cutoff for model adequacy of less than 0.08.

## Results

### Exploratory factor analysis (EFA)

Table 1 shows the Pearson correlation coefficients for all items, with correlations between similarly-phrased items highlighted in bold. Exploratory factor analysis (principal components analysis with Varimax rotation) suggested a two factor solution explaining 59.0% of the total variance in items (factor 1 eigenvalue = 3.9; factor 2 eigenvalue = 3.1). Consistent with the hypotheses of this study, the first factor comprised all of the NP items and the second factor all of the PP items. Table 2 shows the rotated component matrix for all items, illustrating the division between NP and PP items. This two-factor model was further examined by CFA, below (hypothesis 2, model 2).

**Table 1:** Pearson correlations between all items (PP items p1 to p6; NP items n1 to n6)

	p2	p3	p4	p5	p6	n1	n2	n3	n4	n5	n6
p1	**0.43**	**0.41**	**0.47**	**0.43**	**0.39**	0.37	0.38	0.38	0.39	0.40	0.36
p2	-	**0.49**	**0.43**	**0.40**	**0.38**	0.27	0.25	0.34	0.33	0.38	0.39
p3	-	-	**0.36**	**0.47**	**0.38**	0.24	0.24	0.33	0.28	0.36	0.32
p4	-	-	-	**0.50**	**0.41**	0.32	0.37	0.40	0.39	0.38	0.35
p5	-	-	-	-	**0.48**	0.33	0.36	0.42	0.41	0.42	0.41
p6	-	-	-	-	-	0.39	0.40	0.42	0.51	0.48	0.49
n1	-	-	-	-	-	-	**0.58**	**0.54**	**0.57**	**0.51**	**0.44**
n2	-	-	-	-	-	-	-	**0.60**	**0.60**	**0.53**	**0.44**
n3	-	-	-	-	-	-	-	-	**0.60**	**0.60**	**0.54**
n4	-	-	-	-	-	-	-	-	-	**0.69**	**0.58**
n5	-	-	-	-	-	-	-	-	-	-	**0.71**

**Table 2:** Rotated component matrix for exploratory factor analysis

Item	Factor 1	Factor 2
**n4**: Been feeling unhappy and depressed	*0.82*	0.25
**n2**: Felt constantly under strain	*0.78*	0.16
**n5**: Been losing confidence in self	*0.77*	0.32
**n1**: Lost sleep over worry	*0.75*	0.16
**n3**: Felt couldn't overcome difficulties	*0.75*	0.29
**n6**: Been thinking of self as worthless	*0.68*	0.34
**p3**: Felt capable of making decisions	0.10	*0.76*
**p2**: Felt playing useful part in things	0.16	*0.74*
**p5**: Been able to face problems	0.30	*0.69*
**p4**: Able to enjoy day-to-day activities	0.29	*0.66*
**p1**: Able to concentrate	0.31	*0.64*
**p6**: Been feeling reasonably happy	0.47	*0.52*

### Hypothesis 1: Comparison of the variances of NP and PP items

**Figure 2:**
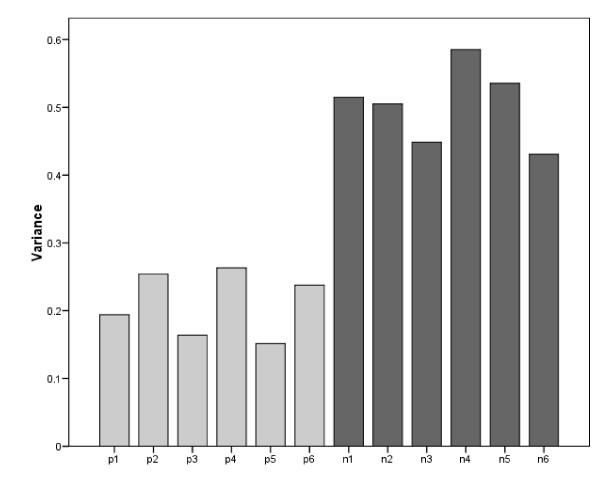
Item variances (PP items p1 to p6; NP items n1 to n6).

Table 3 shows the variances of all items with 95% confidence limits (see also Figure 2 for a graphical representation of the variances of the items). It can be readily seen that the variances of the NP items (median 0.515) were uniformly higher than those of the PP items (median 0.215), with no overlap in the 95% confidence limits. This supports the first hypothesis that the NP items would have increased variances due to additional error variance caused by response bias.

**Table 3:** Variances (95%CL) for PP and NP items

PP items	Variance	95%CL for variance
p5	0.15	0.14, 0.16
p3	0.16	0.16, 0.17
p1	0.19	0.19, 0.20
p6	0.24	0.23, 0.25
p2	0.25	0.24, 0.27
p4	0.26	0.25, 0.27

**NP items**		
n6	0.43	0.41, 0.45
n3	0.45	0.43, 0.47
n2	0.51	0.48, 0.53
n1	0.52	0.49, 0.54
n5	0.54	0.51, 0.56
n4	0.59	0.56, 0.61

### Hypothesis 2: Comparison of models

Table 4 shows the goodness-of-fit measures for all models. Of the previously reported models the best fitting was the Graetz three dimensional model; indeed, of models 1 to 3 this was the only model with an acceptable fit (RMSEA = 0.073, 90%CL (0.069, 0.077); ECVI = 0.301, 90%CL (0.274, 0.331). Model 4 was, however, superior in all fit measures to model 3 (RMSEA = 0.068, 90%CL (0.064, 0.073; ECVI = 0.214, 90%CL (0.191, 0.238)), thus confirming the second hypothesis.

**Table 4:** Fit measures for all models

Model	GFI	AGFI	NFI	CFI	RMSEA (90%CL)	ECVI (90%CL)
1. Unidimensional	0.843	0.774	0.846	0.848	0.125 (0.121, 0.128)	0.867 (0.818, 0.917)
2. Two-dimensional	0.931	0.898	0.927	0.929	0.086 (0.082, 0.090)	0.419 (0.386, 0.454)
3. Three dimensional	0.953	0.928	0.948	0.951	0.073 (0.069, 0.077)	0.301 (0.274, 0.331)
**4. Unidimensional with correlated errors**	**0.969**	**0.938**	**0.965**	**0.967**	**0.068 (0.064, 0.073)**	**0.214 (0.191, 0.238)**

### Exploration of NP response bias

**Figure 3:**
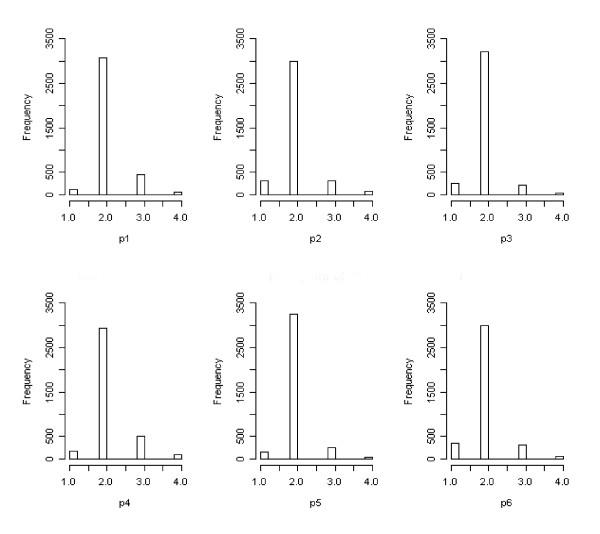
Frequency histograms of PP item responses (items p1 to p6).

**Figure 4:**
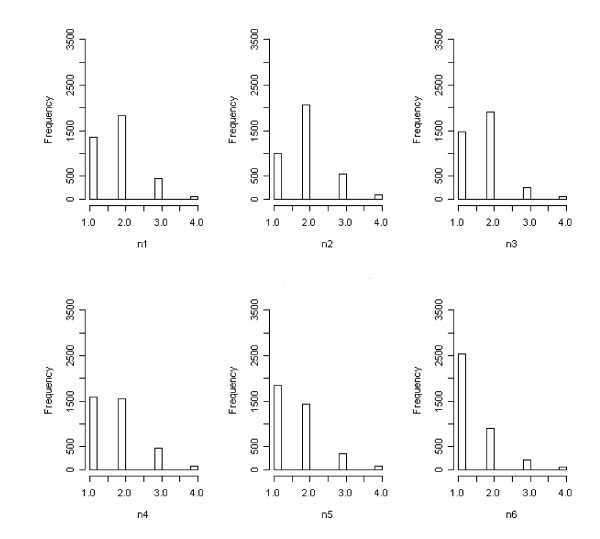
Frequency histograms of NP item responses (items n1 to n6).

Figure 3 shows frequency histograms for the six PP items. It can be seen that most of the respondents indicated the presence of positive mood states by responding on point 2 of the four point Likert type scale, indicating that these positive mood states were experienced with the 'Same' frequency as usual (i.e. a lack of pathology was indicated by a PP item score of 2). In contrast, Figure 4 shows the frequency histograms for the six NP items. It can be seen that the same respondents indicated an absence of negative mood states for items n1 to n4 with a response of either 1 or 2 on the four-point scale. These correspond to responses of 'Not at all' and 'No more than usual'. Hence, respondents wishing to indicate an absence of negative mood states were forced to choose between two response options due to the ambiguous wording, and the distribution was split between the two scale points. Items n5 and n6 correspond to the extreme negative mood states of 'Losing confidence' and 'Feeling worthless', for which the response 'No more than usual' makes little sense in the absence of such mood states. Hence for these items there was less ambiguity and correspondingly more respondents opted for point 1 of the scale.

The additional variance in the NP items may therefore be explained: the ambiguous response options for the NP items shift many responses from point 2 to point 1, increasing the scale variance. The appearance of two factors in previous studies is also explained in this way, since the shift in the distribution results in the NP items being more correlated with each other than with the PP items (see Table 1: median NP inter-item correlation *r *= 0.58; median PP inter-item correlation *r *= 0.43; median NP inter-item correlation partialling PP items *r*_*p *_= 0.43), thus generating a spurious two factor solution comprising NP and PP items. The Graetz three factor model may also be attributed to this mechanism, with an additional factor generated by the two least ambiguous NP items (n5 and n6 correspond to the factor of 'Losing confidence' reported by Graetz).

## Discussion

Simple exploratory factor analysis replicated previous studies, suggesting a two factor solution comprising exclusively NP and PP items. Confirmatory factor analysis, however, suggested that this model was not a good fit for the data (RMSEA = 0.086; 90% CL = 0.082, 0.090). The best fitting model was the hypothesised model with response bias on the NP items (RMSEA = 0.068; 90% CL = 0.064, 0.073). Interpreting the simple EFA solution as signalling the existence of substantive constructs (for example, factor 1 as 'depression' and factor 2 as 'social dysfunction') was therefore not warranted by the data, and would represent an example of the methodological error of reification. That the best fitting model was the model incorporating method effects suggests that the most parsimonious interpretation of the data is that the GHQ-12 is largely or wholly unidimensional, but with substantial response bias on the NP items. While this finding is in accord with the intended function of the GHQ-12 (as a unidimensional, non-specific index of psychiatric morbidity), it should be remembered that the accuracy of measurement depends on low measurement error and the absence of substantial response bias. One assumption of this study is that the random measurement error is approximately equal across all items, and that any difference in variance between NP and PP items is largely due to response bias. Although this assumption is confirmed in the analysis of the variance of the items and the pattern of inter-item correlations, it is of course also consistent with a genuine two or three factor solution in which the factors themselves have different variances. This alternative explanation is, however, even less parsimonious, since it hypothesises not only that multiple factors represent substantive constructs, but that those constructs differ in variance along the same lines as the NP and PP items. Further, since no previous study has hypothesised in advance that (a) multiple factors exist and (b) they will differ significantly in their variance, justification of this kind would be ex post facto, in contrast with the a priori hypothesis tested in this study.

In contrast to the causal mechanisms proposed in previous studies, the response bias seems not to result from inattention, lack of education or aversion to negative phrasing, but from the ambiguous response frame used for the NP items. Respondents wishing to indicate an absence of a negative mood state are faced with two possible choices: 'Not at all' or 'No more than usual'. For respondents not suffering from psychiatric problems, either of these options reflects their current status. This ambiguity increases the variance of the NP items and adds a correlated component to the error term for these items, thus generating spurious factors. It remains to be seen if this method effect applies in populations with a higher prevalence of psychiatric disturbance.

## Conclusion

While many studies report the factor structure of the GHQ-12 as two- or three-dimensional, it appears that these are spurious findings resulting from a neglect of method effects, specifically a response bias on the items expressing negative mood states. This is turn seems to be due to the ambiguous response options for indicating the absence of negative mood states. While the results presented here are consistent with the GHQ-12 being unidimensional as originally intended, the presence of substantial response bias on the negatively phrased items required careful attention in the analysis of clinical epidemiological data. This is especially true of the assessment of the reliability of the GHQ-12, since the computation of reliability coefficients such as Cronbach's alpha assumes no such bias, and the resulting reliability may be over- or under-estimated.

## Competing interests

The author declares that they have no competing interests.

## Authors' contributions

MH is the sole author
